# Traffic-Related Air Pollution and Parkinson’s Disease in Denmark: A Case–Control Study

**DOI:** 10.1289/ehp.1409313

**Published:** 2015-07-07

**Authors:** Beate Ritz, Pei-Chen Lee, Johnni Hansen, Christina Funch Lassen, Matthias Ketzel, Mette Sørensen, Ole Raaschou-Nielsen

**Affiliations:** 1 Department of Epidemiology, University of California at Los Angeles School of Public Health, Los Angeles, California, USA; 2 Department of Neurology, School of Medicine, University of California at Los Angeles, Los Angeles, California, USA; 3 Department of Health Care Management, College of Healthcare Administration and Management, National Taipei University of Nursing and Health Sciences, Taipei, Taiwan; 4 Danish Cancer Society Research Center, Danish Cancer Society, Copenhagen, Denmark; 5 Department of Environmental Science, Aarhus University, Roskilde, Denmark

## Abstract

**Background:**

Very little is currently known about air pollutants’ adverse effects on neurodegenerative diseases even though recent studies have linked particulate exposures to brain pathologies associated with Parkinson’s and Alzheimer’s disease.

**Objective:**

In the present study, we investigated long-term exposure to traffic-related air pollution and Parkinson’s disease.

**Methods:**

In a case–control study of 1,696 Parkinson’s disease (PD) patients identified from Danish hospital registries and diagnosed 1996–2009 and 1,800 population controls matched by sex and year of birth, we assessed long-term traffic-related air pollutant exposures (represented by nitrogen dioxide; NO_2_) from a dispersion model, using residential addresses from 1971 to the date of diagnosis or first cardinal symptom for cases and the corresponding index date for their matched controls. Odds ratios (ORs) and 95% confidence intervals (CIs) were estimated with logistic regression, adjusting for matching factors and potential confounders.

**Results:**

We found ambient air pollution from traffic sources to be associated with risk of PD, with a 9% higher risk (95% CI: 3, 16.0%) per interquartile range increase (2.97 μg/m^3^) in modeled NO_2_. For participants living for ≥ 20 years in the capital city, ORs were larger (OR = 1.21; 95% CI: 1.11, 1.31) than in provincial towns (OR = 1.10; 95% CI: 0.97, 1.26), whereas there was no association among rural residents.

**Conclusions:**

Our findings raise concerns about potential effects of air pollution from traffic and other sources on the risk of PD, particularly in populations with high or increasing exposures.

**Citation:**

Ritz B, Lee PC, Hansen J, Funch Lassen C, Ketzel M, Sørensen M, Raaschou-Nielsen O. 2016. Traffic-related air pollution and Parkinson’s disease in Denmark: a case–control study. Environ Health Perspect 124:351–356; http://dx.doi.org/10.1289/ehp.1409313

## Introduction

Air pollution is ubiquitous, and particulate exposure levels are currently rising to unprecedented levels in some emerging economies, with traffic sources being major contributors. Air pollutants’ adverse effects on respiratory and cardiovascular health are well documented (Andersen et al. 2012; Anderson et al. 2012; Lim et al. 2012; Shah et al. 2013), yet very little is currently known about the effects they may have on the aging brain. Parkinson’s disease (PD) is the second most common neurodegenerative disorder, engendering great human costs in aging populations (Huse et al. 2005), and recent evidence suggests that air pollution may act on biologic pathways contributing to PD. Specifically pathologic studies of the human brain and some animal experiments reported neuroinflammation, oxidative stress, and dopamine system–related neurotoxicity with air pollution exposures (Block and Calderón-Garcidueñas 2009; Calderón-Garcidueñas et al. 2010; Cannon et al. 2009; Levesque et al. 2011; Yamin et al. 2003).

Previously, researchers found neuropathological lesions in feral dogs living in Mexico City, as well as inflammation of the olfactory bulb and deficits in olfaction in Mexican children residing in highly polluted areas (Calderón-Garcidueñas et al. 2008a, 2010). It has recently been proposed that the olfactory bulb may provide an anterograde entry passage for pathogens and xenobiotics—including particles—along the olfactory tract, that is, a nose-to-brain route that bypasses the protective blood–brain barrier (Hawkes et al. 2009; Lucchini et al. 2012). Interestingly, the Mexican researchers also detected α-synuclein neuronal aggregates, characteristically found in Lewy bodies of patients with PD, as well as an increased number of oxidative stress markers in brainstem nuclei of autopsied young people who died suddenly and had been exposed to Mexico City’s air pollution (Calderón-Garcidueñas et al. 2008b). Furthermore, misfolded α-synuclein proteins were recently found to be transmissible within the brain, that is, to spread from affected to unaffected neurons by seeding misfolding of proteins (Desplats et al. 2009; Luk et al. 2012; Mougenot et al. 2012), a possible mechanism by which PD pathology (protein agglomeration) may reach the brain through the olfactory tract.

It is well known that one of the early preclinical features of PD observed in almost all cases is the loss of the sense of smell (Doty 2012a, 2012b; Levesque et al. 2011), and Braak’s neuropathological staging of PD suggests that olfactory bulb neurons are among the first to display Lewy body pathology characteristic of PD (Braak et al. 2004). Airborne ultrafine (nanosized) particles have been shown to translocate to mitochondria after endocytosis, to produce reactive oxygen species, and to pass through the blood–brain barrier (Braak et al. 2004; Oberdörster and Utell 2002; Oberdörster et al. 2004). Major sources of population exposure to nanoparticles are internal combustion processes and traffic exhaust (Oberdörster and Utell 2002). Although population monitoring of ultrafine particles is not available, land use regression (LUR) modeling that relies on easier to measure gaseous indicators such as nitrogen oxides (NO_x_) and nitrogen dioxide (NO_2_) have been shown to capture the fine spatial variation of pollutant mixtures from traffic well (Su et al. 2009).

Epidemiologic research of air pollution and dopaminergic neurodegeneration is just beginning. A cross-sectional study investigating manganese in air pollution suggested a small increase in PD prevalence and lower age at onset (Finkelstein and Jerrett 2007). An ecologic study of urban counties linked industrial emissions of copper and manganese recorded by the U.S. Environmental Protection Agency (EPA) to PD cases identified from Medicare records (Willis et al. 2010). Most recently researchers relied on the Harvard Nurses’ Health cohort and assessed exposures by modeling levels of *a*) metals that are considered air toxics (Palacios et al. 2014b) and *b*) fine and coarse particulate matter (Palacios et al. 2014a); these studies, however, did not find associations with PD. Here we present for the first time data that associate PD with long-term (1971 onward) and fine scale–modeled exposure to NO_2_ used as a marker of traffic-related air pollution mixtures in a nationwide study in Denmark.

## Methods

*Study population.* PD patients were identified from the Danish National Hospital Register (Lynge et al. 2011) between 1996 and mid-2009, and all were treated at 10 large neurological centers in Denmark. This register has kept computerized records of all hospitalized patients in Denmark since 1977 and also from outpatient clinics after 1994, including information on the patient (name plus the unique 10-digit personal number that is applied to all residents in Denmark) and their primary as well as other diagnoses (Lynge et al. 2011). As described in more detail (Wermuth et al. 2012), medically trained research staff supervised by a movement disorder specialist reviewed medical records we obtained from the treating hospitals/outpatient clinics and rigorously applied standard criteria to establish an idiopathic PD diagnosis (Hughes et al. 1992). The diagnosis was based on all medical record information available at time of record retrieval, including notes from private practitioners, and we required the presence of at least two of the following symptoms: resting tremor, bradykinesia, rigidity, and asymmetrical onset. We also evaluated response to treatment with levodopa, signs of dementia and their timing, early falls, severe symptomatic dysautonomia, and sudden symptom onset, supra-nuclear gaze palsy, hallucinations unrelated to medication, and records for computed tomography scans, DaTscans (Dopamine transporters Scan), or magnetic resonance scans. The occurrence of the first cardinal (motor) symptom noted on the medical record or—if missing—the first known date of hospitalization/outpatient clinic visit due to PD was the reference date for calculating time-dependent exposures for cases and matched controls. From a list of 2,762 initially eligible patients, we removed 179 subjects without idiopathic PD (iPD) according to medical records we received before interview, 20 without medical records to confirm diagnoses, and 497 (21.3%) who declined to participate. We further excluded 238 cases after interview when the medical record review determined that they did not suffer from iPD, leaving 1,828 confirmed iPD cases.

Initially, for each case we sampled 5–10 potential controls randomly from the Danish Central Population Register, which covers historical information, including names, dates of birth, death, and immigration on all residents in Denmark. We aimed to enroll and interview one control from each matched set. Thus, whenever a contacted control was willing to participate, no other potential control from the same set was contacted. Controls were required to not have PD based on Danish National Hospital Register records before the date their respective case received a PD diagnosis and to be alive for interview. They were assigned the same index date as their respective case. Of 3,626 eligible controls initially contacted, 1,909 (52.6%) completed an interview. All participants were interviewed between January 2008 and December 2010 to obtain information on possible confounding variables including education, tobacco-smoking history, and family history of PD.

Written informed consent was obtained from all subjects, and the study protocol was approved by the UCLA Institutional Review Board and by the Danish Data Protection Agency and the ethics committee for the Copenhagen Region (H-D-2007-0009).

*Address geocoding and air pollution exposure assessment.* We retrieved all addresses of study participants from 1 January 1971 onward from the Central Population Registry, relying on the unique personal identification number for Danish citizens and including the dates of moving to and leaving each address before index date. Addresses, identified by municipality code, street code, and house number, were linked to the Danish Address Register to obtain geographical coordinates at the front door of the house. The precision of the geographical coordinates was high, lying within 5 m for most addresses, and we successfully geocoded and estimated air pollution exposures at 88% of all addresses. The geocodes in OSAK (Official Standard Addresses and Coordinates; http://www.addresse-info.dk/)—the address/GIS registry used to translate address codes to geocodes—refer to the middle of a building. If there are multiple addresses in one building, the geocodes reflect the location of the front door of the specific address. The accuracy in OSAK is reported as 95–98% of the geocoding having accuracy better than 10 m and for most of these the accuracy is better than 5 m. For 2–5% of the addresses, geocodes are calculated for the center point of the land parcel registered (the piece of land on which the building is situated). Thus the accuracy depends on the size of the parcel; for single-family houses, the precision will typically be better than 50 m.

We employed a GIS-based dispersion modeling system (AirGIS; http://envs.au.dk/en/knowledge/air/models/airgis/) to estimate subjects’ exposure to well established surrogates of traffic-generated air pollution [nitrogen dioxide (NO_2_), nitrogen oxides (NO_x_) carbon monoxide (CO)] averaging over the period starting in 1971 up to the participant’s index date. Specifically, we calculated air pollution estimates for each address as the sum of *a*) local air pollution from street traffic, calculated from traffic intensity and type, emission factors for the car fleet, street and building geometry and meteorology; *b*) urban background, calculated from data on urban vehicle emission density, city dimensions and building heights; and *c*) regional background, estimated from trends at rural monitoring stations and from national vehicle emissions. With the geocode of an address and a specified year as the starting point, the AirGIS system automatically generates street configuration data for the street pollution model, including street orientation, street width, building heights in wind sectors, amount of traffic, speed, and type as well as other required data.

We estimated historical traffic-related air pollutant exposures based on AirGIS estimates for hourly air pollution concentration for NO_2_, NO_x_, and CO at each address and calculated average concentrations between 1 January 1971 and the index date for each participant. We included in analyses only participants for whom the residential addresses were known and geocoded, and this allowed us to generate air pollution measures for ≥ 80% of the period between 1 January 1971 and the index date; 6% of cases and 5% of controls did not fulfill this criterion—that is, a total of 1,696 (93%) cases and 1,800 (94%) controls were included in our analyses. For those missing pollutant data for < 20% of the period, we calculated exposure averages based only on days with nonmissing data.

*Statistical methods.* Exposure measures for the air pollutants were modeled as continuous (per interquartile range increase). We also investigated the shape of the exposure–response function between air pollution and PD using percentiles of exposure and splines with 2 knots (smoothers). Exposure lagging—excluding exposures that occurred within certain periods (5 or 10 years) before disease onset or interview—were explored to address concerns that exposures in years immediately preceding the PD diagnosis may not be etiologically relevant. Lag times of 5 and 10 years cover the latency period range most often suggested for pre-motor PD (Siderowf and Lang 2012). To include the largest possible number of cases and controls and to conduct stratified analyses, we broke the matches and used unconditional logistic regression models to obtain odds ratios (ORs) and 95% confidence intervals (CIs). We selected potential confounding variables using causal diagram methods as well as the > 10% change in effect estimate criteria (Mickey and Greenland 1989). Thus, in our models we adjusted for the matching variables, birth year, sex, and age at index date, and also for other potential confounding risk factors obtained in interviews, such as education (basic, vocational, and higher education), smoking [smoking status (never, former, current smoker) and pack-years of smoking], family history of PD (first-degree relative with PD history diagnosed by a doctor; yes/no), and job history of air pollution–related exposures (i.e., any history of professional truck, tractor, or bus driving; or having worked as traffic police officer, gas station attendant, mechanic, or asphalt worker). In adjusted models, subjects with missing covariate data were excluded. Controls retained the index date they were originally assigned even after matching was broken. Smoking status and all other exposures were defined as lifetime exposure before index date. In sensitivity analyses, we examined air pollution effects by smoking status, sex, age at index date for cases and controls before or after age 60 years, and location of the residence (Copenhagen and suburbs, provincial cities, rural towns and villages). We also investigated effects only in those diagnosed most recently between 2005 and 2009. We assessed effect modification in stratified analyses and by entering product terms into our logistic regression models. The interaction *p*-values were based on a log likelihood ratio test. A one-sided *p*-value < 0.05 was considered statistically significant.

## Results

The PD patients included in this study were on average 62 years of age at first symptom onset or diagnosis, more were male than female (60–40%), half had received vocational training and about a quarter had a higher level of education; in all of these characteristics, controls and cases were quite similar (Table 1). However, PD patients were more often never and former smokers and also somewhat less likely to be born and living in Copenhagen than controls. Our exposure estimates covered an average of 30.7 years of traffic-related air pollution exposure. We focused on results for NO_2_ as a marker of traffic pollution mixtures, because it is most commonly reported in air pollution research; NO_2_ was highly correlated with NO_x_ and CO (Pearson correlation coefficients of 0.92 and 0.81, respectively; the Pearson correlation coefficient for NO_x_ and CO was 0.85).

**Table 1:** Demographic characteristics of the study population by PD status.

Characteristic	Cases (*n* = 1,696)	Controls (*n *= 1,800)
Age (years)^*a*^	61.65 ± 9.50	61.57 ± 9.65
Sex
Female	688 (40.6)	733 (40.7)
Male	1,008 (59.4)	1,067 (59.3)
Education^*b*^
Basic education and high school (7–12 years)	402 (23.7)	410 (22.9)
Vocational training (10–12 years)	822 (48.6)	889 (49.6)
Higher education (≥ 13 years)	469 (27.7)	494 (27.5)
Smoking history^*b*^
Never	841 (49.7)	642 (35.9)
Former	711 (42.1)	788 (44.0)
Current	139 (8.2)	360 (20.1)
Place of birth^*b*^
Copenhagen and suburbs	349 (21.2)	473 (27.0)
Provincial cities	926 (56.4)	903 (51.7)
Rural	367 (22.4)	373 (21.3)
Place of residence 20 years before diagnosis^*b*^
Copenhagen and suburbs	435 (25.7)	544 (30.3)
Provincial cities	959 (56.6)	953 (53.1)
Rural	300 (17.7)	299 (16.6)
Place of residence at diagnosis^*b*^
Copenhagen and suburbs	435 (25.7)	544 (30.3)
Provincial cities	959 (56.6)	953 (53.1)
Rural	300 (17.7)	299 (16.6)
Family history of PD
None	1,460 (86.1)	1,707 (94.8)
At least one first-degree relative diagnosed with PD	236 (13.9)	93 (5.2)
NO_2_ (μg/m^3^; 1971 to censoring)	13.83 ± 4.22	13.59 ± 3.57
Values are mean ± SD or *n* (%). ^***a***^Age at first cardinal symptom of PD patients and their matched controls. ^***b***^Missing: education (*n* = 10), smoking (*n* = 15), place of birth (*n* = 105), place of residence 20 years before diagnosis (*n* = 6), and place of residence at diagnosis (*n* = 81).		

**Figure 1:**
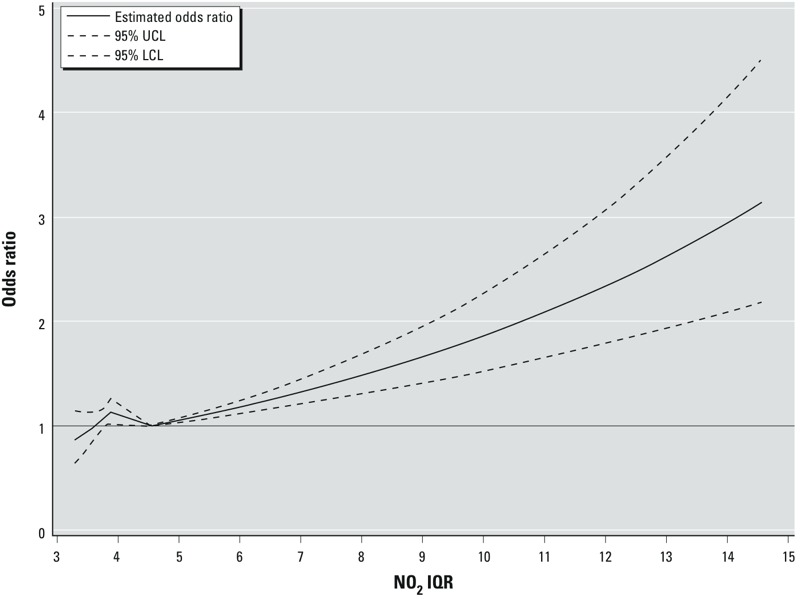
ORs and 95% CIs based on a spline model (with 2 knots) of NO_2_, with each unit on the *x*-axis corresponding to a multiple of the IQR (2.97 μg/m^3^) over the estimated range of exposures (9.80–43.26 μg/m^3^, corresponding to 3.3 and 14.6 IQR units, respectively), relative to the mean NO_2_ concentration (13.71 μg/m^3^, corresponding to 4.6 IQR units). Abbreviations: LCL, lower confidence limit; UCL, upper confidence limit. Model adjusted for age at diagnosis or index date, sex, education, smoking status (never, former, current smoker), pack-years of smoking, family history of PD, year of birth, and job history.

Concentrations of NO_2_ in Copenhagen (mean, 16.83 μg/m^3^) were higher than concentrations in provincial cities (mean, 12.63 μg/m^3^) and rural areas (mean, 12.11 μg/m^3^) (Table 2). For each interquartile range (IQR) increase (2.97 μg/m^3^) in long-term exposure to NO_2_, we estimated a 9% increase in PD risk (OR = 1.09; 95% CI: 1.03, 1.16), adjusting for year of birth, sex, age at first cardinal symptom, education, smoking, family history of PD, and job history of indicators for exposure to traffic pollution (Table 3). Using categories of exposure based on percentiles, we estimated an adjusted OR of 1.08 (95% CI: 0.90, 1.30) for exposures in the 25th–75th percentile and an OR of 1.22 (95% CI: 0.99, 1.51) above the 75th percentile for NO_2_ compared with the < 25th percentile, suggesting that our results were not unduly influenced by the highest exposures; yet NO_2_ exposures above the 95th percentile suggested a strong association (OR = 1.92; 95% CI: 1.32, 2.80) based on 103 cases and 72 controls in this category. Spline models with 2 knots (smoothers) showed an overall increase in risk with increasing exposure above the mean NO_2_ concentration, but with some scatter in the lower end of the exposure range and imprecise estimates in the upper part of the range due to sparse data (Figure 1). ORs were comparable between men and women (Table 3). Associations were slightly stronger in current smokers than in never and former smokers, but the interaction with smoking status was not significant overall (*p*-interaction = 0.31). Point estimates appeared somewhat larger for patients experiencing first symptoms or being diagnosed before age 60 years compared with those diagnosed at older ages, but CIs largely overlapped (interaction *p* = 0.40). Restricting our analyses only to participants with diagnosis or index dates from 2005 to early 2009 (*n* = 1,351) had little effect on the OR, though the estimate was less precise (adjusted OR = 1.08; 95% CI: 0.95, 1.24). None of the differences in estimates presented in Table 3 for different subgroups were formally statistically significant when testing for interactions in our regression models.

**Table 2:** Descriptive statistics of average residential air pollution exposures.

Pollutants	Mean ± SD	IQR	Percentile					
			Minimum	25th	50th	75th	95th	Maximum
Place of residence at index date overall
NO_2_ (μg/m^3^)	13.71 ± 3.90	2.97	9.80	11.35	12.24	14.32	21.44	43.26
NO_x_ (μg/m^3^)	21.00 ± 13.02	7.10	13.46	14.73	16.49	21.83	42.12	181.55
CO (mg/m^3^)	0.55 ± 0.16	0.12	0.36	0.46	0.50	0.58	0.82	2.34
Place of residence at index date by place
Copenhagen and suburbs
NO_2_ (μg/m^3^)	16.83 ± 5.23	6.31	10.65	13.02	15.35	19.33	27.11	43.26
Provincial cities
NO_2_ (μg/m^3^)	12.63 ± 2.46	1.99	9.80	11.27	11.83	13.26	16.72	38.62
Rural
NO_2_ (μg/m^3^)	12.11 ± 1.79	1.40	10.64	11.11	11.51	12.51	15.24	29.54

**Table 3:** Associations [OR (95% CIs)] between long-term traffic-related air pollution exposure*^a^* and Parkinson’s disease.

Model	Odds ratio (95% CI)			
	*n* (cases/controls)	Crude^*b*^ OR (95% CI)	Adjusted model^*c*^ OR (95% CI)	*p* for interaction^*d*^
All	1,696/1,800	1.05 (1.00, 1.11)	1.09 (1.03, 1.16)
< 25th percentile	418/440	1.00	1.00	0.06^*e*^
25th to < 75th percentile	849/915	0.98 (0.83, 1.16)	1.08 (0.90, 1.30)
≥ 75th percentile	429/445	1.02 (0.85, 1.24)	1.22 (0.99, 1.51)
Diagnosed from 2005 to early 2009	665/686	0.98 (0.90, 1.07)	1.08 (0.95, 1.24)
Lagging exposures 5 years	1,695/1,800	1.02 (0.98, 1.06)	1.07 (1.02, 1.12)
Lagging exposures 10 years	1,691/1,796	1.02 (0.98, 1.07)	1.08 (1.03, 1.13)
Sex				0.83
Male	1,008/1,067	1.06 (0.99, 1.13)	1.09 (1.01, 1.18)
Female	688/733	1.04 (0.96, 1.13)	1.09 (1.00, 1.19)
Smoking status				0.31
Never	841/642	1.07 (0.98, 1.16)	1.07 (0.98, 1.17)
Former	711/788	1.06 (0.98,1.15)	1.09 (1.00, 1.18)
Current	139/360	1.09 (0.96, 1.23)	1.23 (1.03, 1.46)
Age at first cardinal symptom (years)				0.40
< 60	691/735	1.09 (1.01, 1.18)	1.16 (1.06, 1.27)
≥ 60	1,005/1,065	1.02 (0.95, 1.09)	1.06 (0.98, 1.14)
Place of birth				0.41
Copenhagen and suburbs	349/473	1.11 (1.02, 1.21)	1.16 (1.05, 1.27)
Provincial cities	926/903	1.10 (1.01, 1.20)	1.16 (1.05, 1.29)
Rural	367/373	1.03 (0.89, 1.20)	0.99 (0.83,1.19)
Place of residence at index date (diagnosis of case)				0.14
Copenhagen and suburbs	435/525	1.16 (1.08, 1.25)	1.21 (1.11, 1.31)
Provincial cities	959/908	1.09 (0.98, 1.22)	1.10 (0.97, 1.26)
Rural	300/288	0.93 (0.71, 1.22)	0.93 (0.68, 1.27)
^***a***^Per 2.97-μg/m^3^ increase. ^***b***^Unconditional logistic model adjusted for age at diagnosis or index date, sex, and year of birth. ^***c***^Unconditional logistic model adjusted for age at diagnosis or index date, sex, education, smoking status (never, former, current smoker), pack-years of smoking, family history of PD, year of birth, and job history. ^***d***^Interaction *p*-values were based on a log likelihood ratio test. ^***e***^*p* for trend.				

Associations were similar in size for those born in Copenhagen and its suburbs and provincial towns, but the association with the same increase in NO_2_ was null for those born in rural areas (Table 3). The pattern was similar when stratifying by place of residence at the diagnosis or index date (Table 3) or 20 years before the diagnosis or index date (results not shown). Results did not change when we lagged NO_2_ by 5 and 10 years before the diagnosis/index date (Table 3). Finally, associations were similar for IQR increases in CO (adjusted OR = 1.13; 95% CI: 1.06, 1.21 for a 0.12-ppm increase) and NO_x_ (adjusted OR = 1.06; 95% CI: 1.02, 1.11 for a 7.10-ppm increase).

## Discussion

To our knowledge, this is the first study to suggest that ambient air pollution due to traffic-related sources increases the risk of PD. This association was observed among those who were born or living in Copenhagen or provincial towns, whereas rural residents seemed to not be at higher risk according to our modeled air pollution measures. Also, associations were positive whether participants lived in these places at birth or 20 years before or at time of diagnosis. Estimated effect sizes were largest for those living in Copenhagen.

Air pollution including that from traffic-related sources is well known to adversely affect human health and has recently been shown to contribute to processes that may damage the brain (Block and Calderón-Garcidueñas 2009; Calderón-Garcidueñas et al. 2008b; Campbell et al. 2005; Hartz et al. 2008; Mills et al. 2009; Schwartz et al. 2005; Sun et al. 2005). In animal experiments, ultrafine particulates from combustion (< 0.1 μm in aerodynamic diameter)—the main contributor to traffic-related air pollution—were shown to reach the brain, cause inflammation, and act as neurotoxins (Elder et al. 2006; Oberdörster et al. 2004; van Berlo et al. 2010). In terms of human experimental studies, studies using autopsied human brain samples found significantly higher cyclooxygenase-2 expression, an inflammatory mediator, in the frontal cortex and hippocampus and greater neuronal and astrocytic accumulation of α-synuclein in neurons, glial cells, and/or blood vessels in subjects with lifelong exposure to high levels of air pollution compared with controls from a relatively clean environment (Calderón-Garcidueñas et al. 2004, 2010). These findings are important because the α-synuclein protein and its aggregates are major components of Lewy bodies, a PD hallmark feature. We reported recently that associations between pesticides and PD were stronger among study participants who carried genetic variants linked to higher expression of the α-synuclein gene (Gatto et al. 2010).

Two of four previous human studies investigating air pollution effects on PD risk focused solely on metals classified as air toxics. A Canadian study (Finkelstein and Jerrett 2007) did not find associations with a land use regression–derived traffic-related pollution measure for NO_2_ but reported small positive associations with PD for manganese, a metal that has been related to a syndrome with Parkinsonian features called “manganism” distinct from idiopathic PD (Rivera-Mancía et al. 2011). The authors relied on administrative records for PD drug use and diagnosis codes, but did not validate PD diagnoses and included prevalent cases. Their study also assessed exposures only for the brief period from 1992 to 1999 during which cases were identified from the database—around the time of diagnosis or even after for prevalent cases. The Medicare beneficiary study in the United States (Willis et al. 2010) used a coarse county-scale exposures model and identified manganese and copper from industrial releases as potential risk factors for PD. Recent publications from the Nurses’ Health Study did not find evidence that any of the investigated metals known as air toxics (Palacios et al. 2014b) or the fine and coarse particulate matter exposures they modeled (Palacios et al. 2014a) increased PD risk. However, the exposure period for which modeled residential air pollutant exposures were available overlapped almost entirely with what is now believed a lengthy (10–20 years) preclinical period of PD (Siderowf and Lang 2012); it did not pre-date this preclinical period. Thus, our exposure model for Denmark covered a much longer and possibly more relevant period of participants’ lifetimes. Furthermore, the EPA hazardous air pollution (HAPs) model the Nurses’ Health Study employed is spatially coarse, too coarse to assess mobile sources locally; and though it captures industry-reported releases of air toxics, it does not provide the same data as a finer-scale air toxics monitoring network (U.S. EPA 2011). The PD risk estimate for a continuous measure of PM_2.5_ in the Nurses’ Health Study was 1.08 (95% CI: 0.81, 1.45) per 10 μg/m^3^, an imprecise estimate that suggests a small positive association.

Although Danish air pollution levels are lower than those reported for Southern and Eastern Europe, they are similar to Western industrial nations in terms of the contributions of vehicular traffic exhaust, which continues to be a major source of air pollution in Denmark (Eeftens et al. 2012). Historical and long-term traffic-related air pollution in Denmark modeled as in our PD study have previously been associated with increased risk of developing lung cancer among nonsmokers and also with brain tumors (*International Classification of Diseases, 7th Revision* code 193) in the Danish Diet Cancer and Health cohort (54,304 participants), which relied on the Danish Cancer Registry to identify cancers between enrollment in 1993–1997 until 2006 and traced their residential addresses from 1971 onward in the Central Population Register (Raaschou-Nielsen et al. 2011a, 2011b).

We were unable to directly measure traffic-related air pollution, but the AirGIS dispersion models we employed provided us with estimates of NO_2_ as indicators for traffic pollution mixtures. The AirGIS model for assessing traffic-related air pollution exposures in Denmark uses various detailed traffic parameters with high temporal and address-level spatial resolution validated in several studies (Berkowicz et al. 2008; Ketzel et al. 2011; Raaschou-Nielsen et al. 2000). For example, the correlation between modeled and measured half-year mean NO_2_ concentrations at 204 locations in the greater Copenhagen area had a coefficient of 0.90 (Berkowicz et al. 2008); and the correlation between modeled and measured 1-month mean concentrations of NO_x_ and NO_2_ over 12 years (1995–2006) on a busy street in Copenhagen (Jagtvej, 25,000 vehicles per day, street canyon) had coefficients of 0.88 and 0.67, respectively. Furthermore, residential addresses from the Danish Address Register enabled us to estimate on average > 30 years of air pollution exposures while accounting for mobility, further reducing exposure misclassification. Our study participants resided throughout Denmark, and this residential diversity provided high variability in exposure from air pollution due to traffic sources. However, we lacked workplace address information and were unable to take into account indoor air quality.

A major strength of the study is our ability to select PD patients and controls from most of Denmark relying on the National Hospital Register that identifies all Danish hospitalized and outpatient clinic patients. Further, health care, including hospitals, is free for all citizens in Denmark, thus avoiding biases from differential access to health care. Also, our controls were identified in a population-based manner from the Central Population Register. We were able to minimize disease misclassification through thorough abstraction of medical records for all hospitalized PD patients (15% of all records of eligible patients revealed a diagnosis others than idiopathic PD) (Wermuth et al. 2012). The participation rate in interviews was high among the patients (80.8%), but more moderate in controls (52.6%). Generally, selection bias could occur if a higher proportion of nonresponding controls than cases were exposed to high air pollution. Through record linkage we learned that twice as many nonparticipants as participants had died as of March 2013 (13.0% vs. 5.9% controls and 38% vs. 18% cases). In addition, slightly more nonparticipants than participants were hospitalized with diseases that may be affected by air pollution, specifically ischemic heart disease (14.8% vs. 13.4% controls and 21.3% vs. 16.1% cases). Thus, we cannot rule out selection (or survivor) bias in our study due to the influence of air pollution–related health status on participation. Male sex, older age, and smoking are strong risk factors for cardiovascular and pulmonary diseases. Thus, if our air pollution associations were attributable to selection bias’s removing more controls who were highly exposed to air pollution with cardiovascular and pulmonary risk factors, we would have expected air pollution to be more strongly associated with PD in sensitivity analyses for such higher risk groups, that is, smokers, older, and male subjects. However, we found little evidence for this in our subgroup analyses; we found no differences for traffic-related air pollution estimates between men and women, slightly stronger effects in current than in former and never smokers, but weaker effects in those diagnosed with PD at an older compared with a younger age (> 60 years). We also did not see any changes in estimated effects when adjusting for occupations related to air pollution exposures, such as drivers (data not shown). Effect estimates for residing in Copenhagen and its suburbs were stronger than for other cities, and we did not identify risk increases with traffic exposures among rural residents. This difference may be related to the low levels and/or low geographical variation in traffic-related air pollution in rural Denmark.

PD patients living in Copenhagen were slightly undersampled by the National Hospital Register; 30.7% of controls but only 25.7% of cases were living in the capital and its suburbs at time of diagnosis. One explanation might be that more patients who live in Copenhagen are treated exclusively by private practitioners who do not report to the hospital registers that capture an estimated 80% of all Danish PD cases (patients who are treated at clinics and hospitals; Wermuth L., personal communication). If we missed cases who lived in less-polluted areas, this could have biased our results away from the null.

In summary, the large sample size of our study together with our exposure assessment techniques covering a very long time span allowed us to conduct an investigation that suggests long-term impacts of air pollution on PD risk. Exposure to traffic-related air pollution affects large populations in most countries and may be rising further in the near future due to worldwide urbanization trends. These human data are supported by results from animal experiments and pathology studies and raise concern given the increase in vulnerable aging populations.
